# Commentary: Correlation between the gut microbiota composition and cognitive frailty: a case-control study in community-dwelling older adults

**DOI:** 10.3389/fnut.2026.1929141

**Published:** 2026-07-31

**Authors:** Nikki Georgiadou, Ola Sultan, Benjamin Howell Lole Harris, Louis John Koizia

**Affiliations:** 1 Imperial College Healthcare NHS Trust, London, United Kingdom; 2 University of Toronto, Toronto, ON, Canada; 3 Cutrale Perioperative and Ageing Group, Department of Bioengineering, Imperial College London, London, United Kingdom; 4 Department of Oncology, University of Oxford, Oxford, United Kingdom; 5 Collaborating Centre for Values-based Practice, St Catherine's College, Oxford, United Kingdom; 6 Institute for Liver and Digestive Health, Division of Medicine, University College London, London, United Kingdom

**Keywords:** aging, cognitive frailty (CF), frailty, gut health, microbiome

We read with great interest the study by Wen et al. (1) which examined the association between gut microbiota composition and cognitive frailty (CF) among community-dwelling older adults using 16S rDNA sequencing of fecal samples (1). The authors should be commended for addressing CF as a distinct clinical syndrome and for investigating an increasingly important area of aging research. As interest grows in the microbiota-gut-brain axis, studies examining the intersection between physical frailty, cognitive impairment and the gut microbiome are timely and clinically relevant. An important challenge now is determining whether observed microbial signatures represent biomarkers of cognitive frailty, mediators of disease or potentially modifiable therapeutic targets.

A particularly interesting finding in this study was the presence of significant differences in microbial community composition despite similar alpha diversity between groups. This observation adds to a growing body of literature suggesting that the biological relevance of the microbiome may lie less in overall diversity and more in specific compositional patterns and functional relationships (2). Additionally, the identification of a 35-genera microbial signature capable of distinguishing individuals with CF from controls further highlights the potential of the microbiome as a source of novel biomarkers in geriatric medicine. However, these findings remain exploratory and require external validation, including assessment in age matched and longitudinal cohorts, before any clinical application can be considered. These findings are especially intriguing given increasing recognition of the microbiome as a potential contributor to mechanisms implicated in cognitive frailty, including chronic inflammation, metabolic dysregulation and impaired gut-brain signaling (3).

However, the findings also raise an important conceptual question. Are these microbial alterations specific to cognitive frailty itself or do they reflect broader biological processes underpinning aging and vulnerability. Cognitive frailty is increasingly recognized as a multidimensional syndrome arising from the interaction of physical health, cognition, nutrition, lifestyle factors and systemic inflammation (4). Many of the taxa identified by Wen et al. (1). have also been associated with frailty, inflammatory states and age related disease more broadly (5). Consequently, distinguishing microbiome features unique to CF from those reflecting accumulated biological risk remains challenging.

This question becomes particularly relevant when considering the baseline characteristics of the study population. Participants with CF were substantially older than controls, with a mean age difference approaching 17 years. The CF group also had significantly lower nutritional status, dietary fiber intake, physical activity, sleep quality and educational attainment. Each of these variables is independently associated with differences in gut microbiome composition as well as cognitive and physical function. This raises the possibility that some observed associations reflect confounding, while others may represent biological mediators or integral components of the cognitive frailty phenotype itself. Distinguishing between these possibilities remains an important challenge.

Age itself warrants particular attention. Aging is accompanied by alterations in microbial composition, increasing inter individual variability and changes in microbial functional potential (6, 7). These changes may influence inflammatory pathways, nutrient metabolism and resilience to physiological stressors, all of which are relevant to frailty and cognitive decline. While adjustment for measured variables is welcome, residual confounding remains possible, particularly within a relatively small exploratory cohort, and careful interpretation is therefore warranted.

Importantly, these limitations should not be regarded solely as methodological limitations or confounders. Rather, they may represent an interconnected biological and behavioral pathway through which the gut microbiome affects frailty. The observation that participants with CF had lower fiber intake and poorer nutritional status may therefore be as informative as the microbial differences themselves. Such findings support the view that microbiome alterations should be interpreted within a holistic framework encompassing diet, lifestyle and healthy aging.

The findings relating to *Roseburia* are especially noteworthy. Reduced abundance of *Roseburia* was observed among participants with CF, consistent with previous reports linking this genus to healthy aging and anti-inflammatory effects (8). *Roseburia* species are important producers of short chain fatty acids, particularly butyrate, which play recognized roles in maintaining intestinal barrier integrity, immune regulation and gut brain communication (8, 9). While causal relationships remain unproven, such observations provide biologically plausible mechanisms through which microbiome alterations could influence both cognitive and physical function. As these observations are based on genus level associations derived from 16S rDNA sequencing, future studies using shotgun metagenomics, metabolomics and direct measurement of short chain fatty acids will be important to confirm the proposed functional mechanisms.

Ultimately, the significance of this work may extend beyond biomarker discovery toward understanding the microbiome as a potentially modifiable pathway linking nutrition, physical function and cognition in later life. Improved understanding of the biological mechanisms underpinning these associations may identify novel therapeutic opportunities, as has been demonstrated in other areas of medicine through mechanism-based therapeutic repurposing (10). Rather than viewing microbial signatures solely as diagnostic tools, future research may reveal opportunities for preventative interventions targeting dietary patterns, microbial metabolism and broader lifestyle factors before the onset of established frailty. This perspective is consistent with growing recognition that biological aging is best characterized using multidimensional biomarkers integrating clinical, functional and molecular domains, an approach increasingly advocated across translational aging research (11).

Wen et al. (1) have made an important contribution to a rapidly evolving field. Their findings support continued investigation of the gut microbiome as part of a systems based understanding of cognitive frailty and healthy aging. Future studies incorporating age matched validation cohorts, longitudinal designs, mechanistic analyses and interventional approaches will help determine whether these promising microbial signatures can ultimately inform risk stratification, prevention strategies and interventions aimed at promoting healthy aging.
